# Unveiling the potential of CLL-1: a promising target for AML therapy

**DOI:** 10.1186/s40364-025-00738-6

**Published:** 2025-02-12

**Authors:** Hamed Soleimani Samarkhazan, Sara Zehtabcheh, Hamideh Rahmani Seraji, Safedin H. Beqaj, Shamim Tayefeh, Mohammad Hossein Mohammadi, Mojtaba Aghaei

**Affiliations:** 1 Student Research Committee, Department of Hematology and Blood Banking, School of Allied Medical Sciences, Shahid Beheshti University of Medical Sciences, Tehran, Iran; 2 Department of Hematology and Blood Banking, School of Allied Medical Sciences, Shahid Beheshti University of Medical Sciences, Tehran, Iran; 3 Department of Hematology and Oncology, Taleghani Hospital, Shahid Beheshti University of Medical Sciences, Tehran, Iran; 4 Mountain View Medical Laboratories, Orange, CA USA; 5 UCLA Immunogenetics Center, Immunogenetics (UIC), 1000 Veteran Ave, Los Angeles, CA 90024 USA; 6 Student Research Committee, Ahvaz Jundishapur University of Medical Sciences, Ahvaz, Iran; 7 Thalassemia & Hemoglobinopathy Research Center, Health Research Institute, Ahvaz Jundishapur University of Medical Sciences, Ahvaz, Iran

**Keywords:** C-type lectin-like molecule-1, Macrophages, Graft-versus-host disease, Immunotherapy, Inflammation, Transplantation, Immunomodulation

## Abstract

Acute myeloid leukemia (AML) remains a formidable blood cancer, despite recent advances in treatment. A significant challenge persists in improving patient outcomes, particularly in addressing relapse and treatment resistance. Identifying new therapeutic targets is critical for advancing AML therapy. C-type lectin-like molecule-1 (CLL-1) has emerged as a promising therapeutic target in AML. This cell surface receptor is highly expressed on AML blasts and demonstrates stable expression throughout disease progression. CLL-1’s consistent presence makes it an ideal candidate for monitoring minimal residual disease (MRD), which is a critical indicator for predicting relapse. Beyond its utility as a diagnostic marker, CLL-1 offers exciting potential in the development of immunotherapies. Emerging strategies, such as CAR-T-cell therapy and antibody-drug conjugates (ADCs), are being investigated to leverage the immune system against CLL-1-expressing AML cells. This review examines the structure, function, and expression patterns of CLL-1 in AML and other hematologic malignancies, providing insights into its role in disease pathogenesis and treatment potential. Exploring CLL-1 as a target for diagnosis, MRD monitoring, and immunotherapy opens new avenues for AML treatment. A deeper understanding of its relationship with AML pathogenesis will aid in the development of targeted therapies, offering hope for improved patient outcomes in the future.

## Introduction

AML, the most common type of acute leukemia in adults, is characterized by its complex nature, heterogeneity, and high level of aggressiveness, involving a wide spectrum of genetic alterations and molecular abnormalities that remain a significant challenge in the fields of hematology and oncology [1, 2]. This disease, identified by the atypical growth of immature myeloid cells, needs a comprehensive strategy for control that involves checking for and keeping track of MRD [2]. The assessment of MRD, which denotes the presence of small numbers of leukemia cells that persist following initial therapy, has become increasingly crucial in clinical decision-making, as it offers valuable insights into the likelihood of disease relapse and informs treatment strategies [3]. In recent years, improvements in supportive therapy and expanded access to allogeneic hematopoietic stem cell transplantation have resulted in improved prognoses for pediatric, adolescent, and adult patients diagnosed with acute myeloid leukemia. However, there is still limited progress in the treatment of this challenging disease in elderly patients [4, 5]. With the recent approval of multiple novel medications (midostaurin, gemtuzumab, ozogamicin, CPX-351, enasidenib, CCX-486, oral decitabine-cedazuridine, quizartinib and ivosidenib) [6, 7], a limited proportion of individuals are anticipated to survive for a period ranging from 2 to 5 years postdiagnosis. This is attributed to the fact that the majority of patients inevitably succumb to the illness or adverse effects of the treatment, underscoring the imperative necessity for efficacious and relatively well-tolerated medications that can improve or supplant current therapeutic approaches.

Leukemic stem cells (LSCs) are becoming increasingly interesting targets for eradication due to our increasing understanding of their role in disease persistence and relapse, as well as our continuous improvement in our understanding of their phenotypic properties [8]. The target antigens or epitopes should ideally be specific to LSCs and malignant cells and should be expressed at low levels in normal cells for diagnostic, prognostic, and therapeutic purposes [9]. With the right treatment approaches in place, the cell surface receptor CLL-1 has become a viable biomarker and possible target for the treatment of AML. Myeloid cells, including those implicated in the development of AML, express the type II type C transmembrane lectin receptor CLL-1 on their surface [10, 11]. Numerous investigations have examined the potential of CLL-1 as a marker for prognosis and diagnosis, as well as a target for cutting-edge therapeutic strategies such as T-cell chimeric antigen receptor (CAR) therapies and antibody‒drug combinations. A better understanding of the complex relationship between CLL-1 and AML pathogenesis will provide new insight into appropriate and targeted therapeutic strategies and increase hope for better outcomes for patients fighting this deadly cancer. Here, we review CLL-1 expression in AML, its potential as a diagnostic and prognostic marker, and its potential future role in tracking detectable residual disease and guiding therapeutic approaches.

## Structure and function of CLL-1

**Fig. 1:**
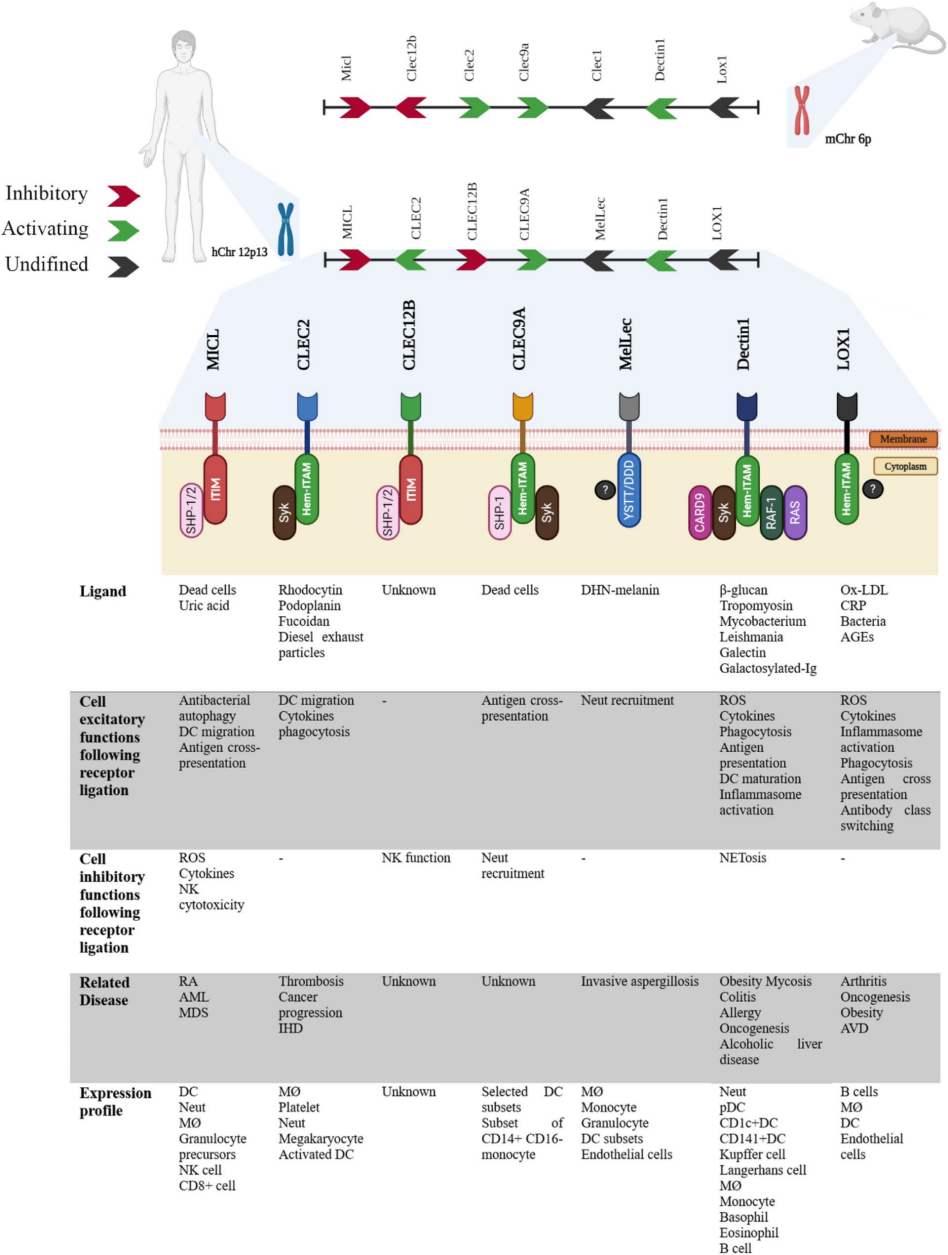
The C-type lectin receptor Dectin-1 cluster. This figure displays the transcriptional direction and genomic organization of the dectin-1 cluster of C-type lectin receptors on human chromosome 12 (green indicates CLR activation, red indicates inhibitory CLR, and black indicates unknown). On mouse chromosome 6, the structure of the murine dectin-1 cluster is comparable. The selected ligands for each receptor are also displayed, along with the pertinent pathological conditions and/or diseases that have been linked to these CLRs. Additionally, the cellular expression profile of the human receptors is presented, with the carbohydrate recognition domain represented by a PAC-Man shape and the cytoplasmic motifs indicated. There are no explicit signaling pathways displayed. AGE, advanced glycation end products; AML, acute myeloid leukemia; MDS; myelodysplastic syndrome; Neut, neutrophil; MØ, macrophage; DC, dendritic cell; BDCA, blood dendritic cell antigen; CD, cluster of differentiation; CRP, C-reactive protein; DHN, 1,8-dihydroxynaphthalene; IHD, ischemic heart disease; MDS, myelodysplastic syndrome; Ox-LDL, oxidized low-density lipoprotein; RA, rheumatoid arthritis. CARD, caspase recruitment domain-containing protein; NET, neutrophil extracellular trap; ROS, reactive oxygen species; RAF, rapidly accelerated fibrosarcoma; Syk, spleen tyrosine kinase

More than 1000 proteins make up a large family of C-type lectin receptors (CLRs), which are proteins with one or more C-type lectin-like domains (CTLDs). This superfamily is categorized into 17 unique subgroups, each of which contributes differently to biological functions, according to phylogenetic analysis and structural configuration [12, 13]. CLRs are crucial for maintaining immune homeostasis because they can recognize a wide variety of endogenic (self) and exogenic (nonself) ligands through CTLDs in a Ca2+-dependent or Ca2+-independent manner. The seven structurally related receptors that make up the Dectin-1 cluster, a subgroup of CLRs, are CLEC-1, CLEC-2, CLEC9A, CLEC12A, CLEC12B, Dectin-1, and LOX-1. These receptors are involved in the regulation of inflammation, infection, and other autoimmune diseases [12, 13] (Fig. 1).

CLL-1, also known as C-type lectin-like 12 A (CLEC-12 A), myeloid inhibitory C-type lectin-like receptor [MICL], CD371, killer cell lectin-like receptor-1 [KLRL1], and dendritic cell-associated lectin 2 [DCAL2], is a type II transmembrane glycoprotein with diverse cellular functions, such as phagocytosis, recognition of pathogens, complement activation, and cell adhesion [10, 11]. The CLL-1 gene in humans is located on chromosome 12p13 within proximity to a cluster housing the C-type lectin/NK gene complex. It is composed of six exons that are responsible for encoding this protein [11, 14]. In mice, this gene is located on chromosome 6 (Table 1) [15].

**Table 1:** CLL-1 features

Protein	Gene name	Gene location	Experssion	Ligand/s	Function	Associated disease
CLL-1, CD371, CLEC12A, hMICL, DCAL-2, KIRL-1	CLEC12A (Human) Clec12a (Mice)	12p13 (Human) 6p (Mice)	DC, Neut, MØ, Granulocyte, Precursors, NK cell, CD8 + cells	Dead cells Uric acid	Necrotic cell Ag, Cross-presentation, SYK inhibition, ↓CXCL1/10, Excessive neutrophil infiltration, ↓ROS and IL-8, ↑Type -I IFN	RA AML MDS

CLEC-12 A, C-type lectin-like 12 A; CLL-1, C-type lectin-like molecule-1; DCAL2, Dendritic cell-associated lectin 2; hMICL, human myeloid inhibitory C-type lectin-like receptor; KLRL1, Killer cell lectin like receptor-1; DC, Dendritic cell; CXCL, Chemokine (C-X-C motif) ligand; ROS, Reactive oxygen species; IL-8, Interleukin-8; IFN, Interferon; AML, Acute myeloblastic leukemia; RA, Rheumatoid arthritis; MDS, Myelodysplastic syndrome; MØ, Macrophage; Neut, Neutrophil; Ag, Antigen

**Fig. 2:**
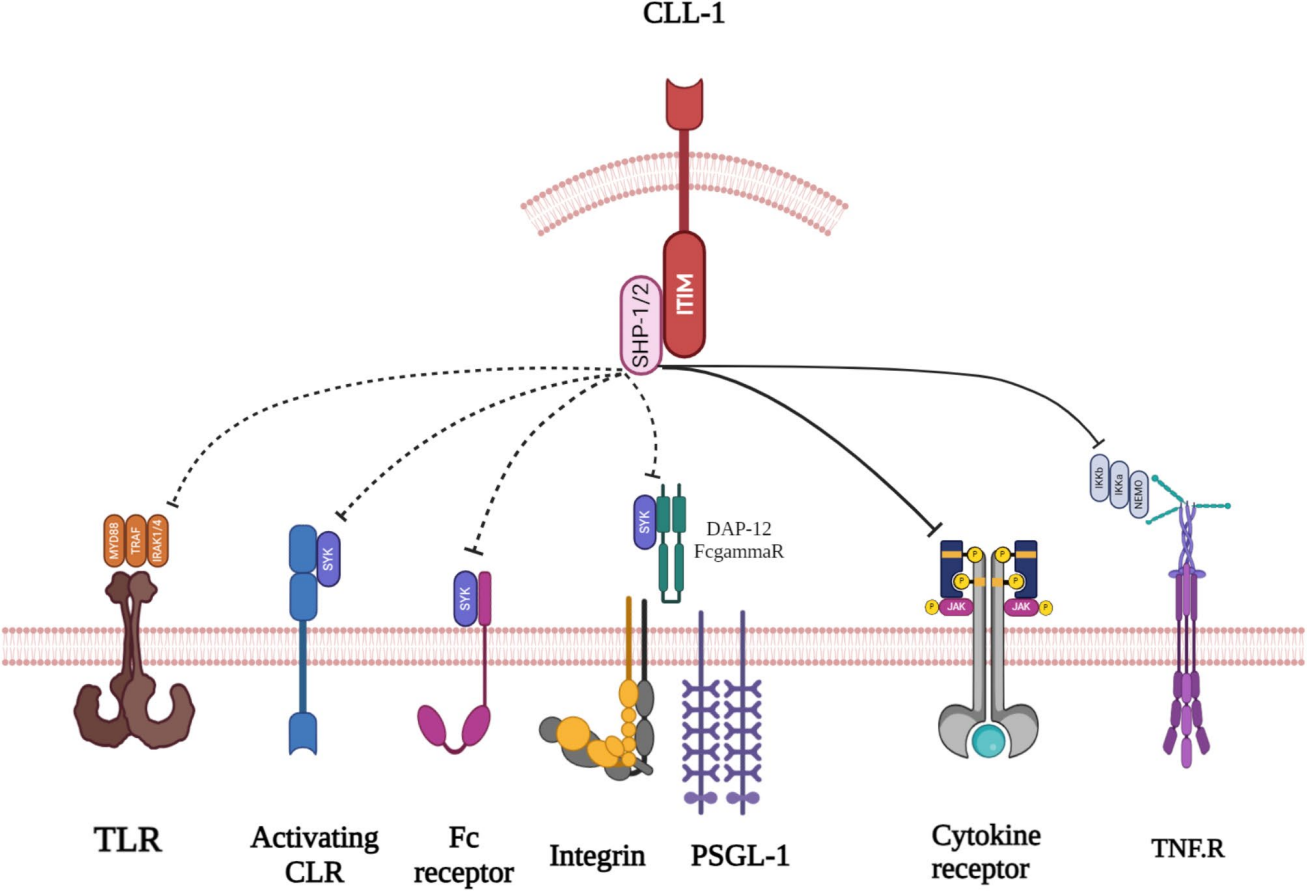
Signal transduction pathways that could be modulated by CLL-1 are illustrated in the figure. The diagram shows the receptors that CLL-1 controls in neutrophils through solid lines, as well as those that it may have the capacity to regulate through dotted lines. This potential regulation is based on the modulation of neutrophil responses induced by MSU and observations made in other myeloid cells. The diagram also highlights the signaling molecules that CLL-1 could inhibit for each receptor. The activation of CLRs, integrins, selectin receptors (such as PSGL-1), and Fc receptors triggers Syk activation either through an ITAM in the receptor itself or through an adaptor protein (DAP12/FcRgamma). Additionally, SHP-1/2 plays a role in dephosphorylating and deactivating Syk, and there is evidence indicating that CLL-1 might impede certain functions associated with these four receptor classes. The sole receptor for which there is direct evidence supporting the inhibition of activation by CLL-1 in neutrophils is the TNF receptor (TNFR). In other myeloid cells, SHP-1/2 dephosphorylates and inhibits IKK and TRAF1/2, suggesting that these proteins may be potential targets in neutrophils. CLL-1 inhibits Toll‐like receptor (TLR) activity in dendritic cells. In other myeloid cells, SHP‐1/2 dephosphorylates and inhibits JAK/STAT proteins linked to cytokine receptors, suggesting a potential mechanism for the inhibition of cytokine signaling by CLL-1. The impact of CLL-1 on other cytokine receptors in neutrophils has yet to be confirmed

The CLL-1 protein is composed of 265 amino acids and possesses a calculated molecular mass of 30.8 kDa, featuring an extracellular segment that structures a singular C-type lectin-like domain housing the six customary cysteines found in C-type lectins. Furthermore, this domain harbors six potential N-glycosylation sites alongside one anticipated O-glycosylation site. The remainder of the protein consists of a projected hydrophobic single transmembrane section in conjunction with a cytoplasmic tail to complete its structural composition. Within this cytoplasmic domain lies an I/VXYXXL immunotyrosine-based inhibition motif (ITIM) and a YXXM motif, which contribute to the functional intricacies of the CLL-1 glycoprotein [11, 16]. The protein tyrosine phosphatases SHP-1 and SHP-2, but not SHIP, are the preferred partners of CLL-1 once they are phosphorylated. According to mechanistic studies using chimeric proteins, CLL-1’s cytoplasmic tail can prevent cellular activation upon ligand-binding stimulation, similar to other ITIM-containing receptors [10, 11] (Fig. 2).

The thymus, heart, kidney, spleen, and bone marrow of mice all contain known CLL-1 self-ligands. The human CLL-1 ligand, however, is not known [12, 17, 18]. Studies on leukemic cell lines and chimeric transfected cells have confirmed that antibody-induced cross-linking of CLL-1 results in internalization of the antigen. CLL-1 selectively cooperates with toll-like receptor signaling pathways or the T-cell signal CD40L to modify the production of cytokines and, consequently, to regulate downstream T-cell activation, according to studies conducted using CLL-1 antibodies in dendritic cells. Since anti-CLL-1 increases the expression of interleukin-6, interleukin-10, MIP-3β, and TNFα and phosphorylates both ERK and p38 MAPK, its role in immunological homeostasis has been considered [11, 19].

In addition to being a native sensor of plasmodial hemozoin and a factor in the development of cerebral malaria, CLL-1 is a well-known specific receptor for the identification of monosodium urate (MSU) crystals from dead cells [12, 20, 21]. It has also been found to be involved in antigen uptake and cross-presentation by human dendritic cells, which leads to strong activation of antigen-activated T cells. CLL-1 plays important roles in the negative regulation of inflammation and positive antiviral responses. Furthermore, CLL-1 is a biomarker of AML cells and is increasingly being used as a target for CAR-T-cell therapy for the treatment of MDS and AML [12, 16]. Functionally, CLL-1 inhibits the production of CXCL1 and CXCL10 as well as the production of ROS and IL8 by neutrophils, which in turn limits the recruitment of neutrophils into tissue after cell damage [17]. Thus, CLL-1 functions as an immune checkpoint that provides a negative feedback mechanism for immunoregulation and shields tissues from an excessive inflammatory response by detecting cell death. Interestingly, a recent study revealed a connection between CLL-1 and the type-I IFN response, which is critical for host immunity against viral infection. Following viral infection, a type-I IFN response is positively activated by MSU released by host dead cells, which in turn amplifies the antiviral immune response [17]. It has also been reported that CLL-1 plays a role in the microbial defense of myeloid cells, specifically in bacterial autophagy [22]. Mice lacking CLL-1 exhibit heightened inflammatory reactions in response to sublethal thymic irradiation, necrotic cells, or MSU. Collagen antibody-induced arthritis (CAIA), which is induced in mice lacking CLL-1 by receiving CLL-1-blocking antibodies, affects these mice more severely. In fact, RA in humans is linked to genetic variations in CLL-1. These findings bolster the regulatory function of CLL-1 in inflammation [15].

## Expression of CLL-1 (physiologically and diseaseally)

### Physiological expression of CLL-1 (in tissue and cells)

Progenitor cells and normal hematopoietic stem cells lack CLL-1 expression (CD34+/CD38- and CD34+/CD33- cell subsets) [11, 23]. CLL-1 is expressed predominantly on myeloid lineage cells, and during differentiation, it is expressed at the common myeloid progenitor (CMP) level. It is highly expressed on granulocyte– myeloid progenitors (GMPs). Megakaryocyte-erythroid progenitors (MEPs) express CLL-1 at significantly lower levels than other progenitor subsets do, according to studies on myeloid progenitors in normal bone marrow [11, 23–25]. Additionally, evidence of functional differences, notably in the CMP and MEP subsets, where CLL-1 positivity favored colony growth of the myelomonocytic lineage, could be obtained via colony-forming cell assays. Granulocytes, basophils, macrophages, monocytes, and CD33dim CD14 + CD16+ (precursor) and CD33bright CD14 + CD16+ (mature) dendritic cells are among the mature myeloid cells that express CLL-1, whereas CLL-1 is not expressed on red blood cells, platelets, NK-, B-, or T cells, or plasmacytoid dendritic cells [15, 23]. Although CLL-1 is expressed in immature monocytes, its expression gradually decreases as the cells differentiate into macrophages [10].

CLL-1 is expressed by most granulocytes and myeloid cells, both CD8 + and CD8-spleen DCs, pDCs, B cells, thymic dual negative and positive cells, CD8 + T cells, and bone marrow NK cells in mice [15]. Peripheral blood leukocytes and bone marrow contain high levels of CLL-1 transcripts, whereas the spleen, fetal liver, heart, colon, placenta, lung, and testis express lower levels of this marker [10]. Solitary splenic tissue exhibited a relatively high positive expression profile in PCR-based analysis of CLL-1 mRNA in 36 tissue types, possibly as a result of local infiltration of neutrophil granulocytes [10, 11, 23]. These findings suggest that expression outside the hematopoietic system is limited. By examining the expression of CLL-1 in human cell lines derived from different sources, only U937, HL-60, THP-1, and MonoMac6 cells of the monocyte/promyelocyte lineage were clearly CLL-1 positive [10, 23].

### Expression of CLL-1 in hematologic disorders

#### The expression of CLL-1 on AML blasts

CLL-1 is commonly found on leukemic blasts in AML. By utilizing multiparameter flow cytometry (MFC), researchers have identified CLL-1 in 77.5 to 92% of AML blasts at the time of diagnosis [11, 23, 26–28]. Research indicates that there is no significant difference in the mean percentage of CLL-1 or MFIR cells among the CD34 + and CD34- subgroups [27, 29–31]. The correlation between marker expression and FAB classification is a matter of debate among researchers, with some stating a significant correlation while others challenging its relevance. For example, Norledin H.E. Darwish et al. reported that the expression of CLL-1 in the M1, M4, M5, and M7 groups was significantly increased (3.5–6-fold), whereas the expression of this marker was relatively lower (2.5-fold) in the M2 group than in the control group [26]. On the other hand, CLL-1 expression was significantly higher (17-fold) in AML FAB M5 patients, indicating a poor prognosis, and patients with AML FAB M4 and M2 expression showed a moderate increase (2.7- and 3.5-fold, respectively). This is associated with a more favorable prognosis [26]. Another study by Gehan M. Hamed et al. revealed that CLL-1 expression varies significantly among FAB subtypes. According to the research results, the M4 subgroup presented the highest mean expression percentage, followed by M1, and the M0 subgroup presented the lowest expression. The highest average MFI was observed in patients with M5, and the lowest was observed in patients with M3 [31].

On the other hand, research conducted by Deena Samir Eissa et al. revealed no notable difference among the AML FAB subtypes [30]. CLL-1 is present in individuals with AML regardless of age, and when adults and children with AML are compared, children present a much greater mean *%* expression and median fluorescence intensity. Additionally, individuals with hepatosplenomegaly following AML exhibit elevated levels of CLL-1 compared with those without this symptom [31].

The presence of CLL-1 is linked to increased levels of type 2 macrophages and monocytes but is associated with decreased numbers of NK cells and regulatory T cells in AML [32]. High levels of CLL-1 are correlated with immune checkpoints and macrophage-related genes [32]. An examination of CLL-1 antigen levels through a quantitative flow cytometry test revealed that the HL-60, U937, and THP-1 myeloid leukemic cell lines have approximately 6 to 17 ^×103^ CLL-1 molecules per cell, whereas peripheral blood monocytes and granulocytes have an average of 10 ^×103^ and 4 ^×103^ molecules per cell, respectively [23]. Analysis of samples obtained during diagnosis and relapse, even from the peripheral blood of patients who are treated with G-CSF, revealed that AML blasts consistently exhibit CLL-1 expression with no variation in antigen density, establishing CLL-1 as a stable marker for monitoring disease progression and detecting MRD; however, it can be inferred that neither chemotherapy nor G-CSF treatment upregulates CLL-1 expression in LSCs [11, 27, 31, 33, 34]. CLL-1 expression percentage and MFIR do not exhibit any notable correlation with sex, age, hemoglobin level, total leukocyte count, platelet count, lactate dehydrogenase levels, lymphadenopathy, status of newly diagnosed untreated patients versus patients in relapse, or different cytogenetic prognostic factors in AML patients [30, 31]. The connection between CLL-1 expression and other AML markers is not fully understood; nevertheless, CLL-1 has shown potential for complementing the classic markers CD33 and CD34 [29].

#### The expression of CLL-1 in other hematologic diseases

##### Myelodysplastic syndrome

Recent research has demonstrated the existence of stem cells in MDS that irregularly present specific antigens, such as IL1RAP, CD25, CD366, and CLL-1, that are potentially linked to high-risk diseases [35]. In a study conducted in 2016 by Marie Toft-Petersen et al., 22 (71%) of the 31 MDS patients examined presented with aberrant CLL-1 expression on CD34+/CD38- cells, regardless of the MDS subtype and IPSS risk group. The results from extended tests on colony-forming cells demonstrated that CD34+/CD38-/CLL-1 + cells were malignant and possessed the ability to regenerate themselves [36]. Nevertheless, CLL-1 could not differentiate normal HSCs from cancerous stem cells since genetic mutations linked to MDS were also present in the CD34+/CD38−/CLL-1 − group [36]. In another study carried out in 2018, Benjamin N. Ostendorf et al. highlighted the aberrant expression of several markers, including ALDH, CLL-1, CD44, and CD47, as specific hematopoietic features in MDS patients with excess blasts. Notably, CLL-1 was found to be expressed at high levels, particularly in various HSPC fractions of MDS-EB patients, including HSCs, multipotent progenitors (MPPs), lymphoid-primed multipotent progenitors (LMPPs), common myeloid progenitors (CMPs), and megakaryocytic-erythroid progenitors (MEPs). The study involved the examination of 20 MDS patients, with aberrant CLL-1 expression mainly observed in 8 patients with MDS with excess blasts and, to a lesser extent, in 12 patients with low-risk MDS [37]. Linde M. Morsink et al. reported that in 18 MDS-EB1/2 patients and the remaining 28 MDS patients, there was no significant difference in overall CLL-1 expression on CD34 + CD38- cells compared with that in the control group (*p* = 0.50). Additionally, the average CLL-1 expression on CD34 + CD38- cells was not significantly different between low-grade MDS patients and MDS-EB patients: 0.0% (range 0–75) and 0.0% (range 0–67), respectively. The majority of MDS cases in this patient cohort did not exhibit CLL-1 expression as a reliable marker for differentiating between normal and aberrant CD34 + CD38- cells, as only 17% of the cases presented CLL-1 expression of 10% or higher [11]. With contradictory findings and limited sample data, CLL-1 seems to be less effective as a diagnostic indicator in MDS than in AML, but more research could provide more insight into the usefulness of CLL-1 in MDS.

##### Myeloproliferative neoplasms

The ability of flow cytometry to count circulating CLL-1 + stem cells can be used to differentiate between MPN phenotypes and potentially track the course of the disease [23, 38]. Myelofibrosis (MF) stem cells exhibit aberrant CLL-1 expression comparable to that observed in AML and MDS. Given the high rate of leukemic transformation observed in MF, it is possible that the CLL-1 + stem cell subset contains cells with true carcinogenic potential or disease-spreading ability. CLL-1 + stem cells are extremely rare in patients with polycythemia vera (PV) and essential thrombocythemia (ET) [38]. A study conducted by L. L. Herborg et al. revealed that CLL-1 was not present on stem cells in NPB or PBSCs, but CLL-1 + stem cells were found to be widely spread across MPN subgroups. Thus, there was a notable increase in abnormal CLL-1 expression in CD34 + CD38- sections from MF (8/10) patients compared with those from PV (4/15) and ET (2/14) patients, and the average percentage of CLL-1 + CD34 + CD38- cells was significantly greater. Surprisingly, a patient with a high percentage of CLL-1 + CD34 + CD38 − cells presented no symptoms and had stable blood tests at the time of sampling but developed aggressive AML within 6 months [38]. On the basis of these results, further research is needed to examine the presence of CLL-1 in MPNs and its impact on disease progression.

##### Acute lymphoblastic leukemia

According to previous data, as expected, CLL-1 is not expressed in T or B-ALL [23, 27]. However, researchers such as Dagmar Schinnerl and her team discovered that in DUX4 + ALL, there is high expression of the CLEC12A gene, which encodes the cell surface protein CLL-1. High expression of CLL-1 is considered to be a unique feature of DUX4-rearranged B-ALL, and DUX4 + is detected in almost all cases. Assessing the immunophenotype of DUX4 + leukemia indicates that the presence of CLL-1 on the cell surface is a remarkably precise indicator for identifying this hard-to-detect genetic subtype. This concept stems from the discovery that, out of 46 cases with DUX4 positivity, 42 had noticeable CLL-1 antigen expression, with three showing weak expression, whereas all other genetic subtypes were mostly negative for it [39].

In a different study, Hind Shakah and colleagues analyzed the presence of CLL-1 at the time of diagnosis in 238 individuals diagnosed with B-ALL. Among these patients, 20 (8.4%) tested positive for CLL-1. On the basis of the results of this research, higher CLL-1 positivity was linked to being diagnosed at an older age, having increased CD34 and CD38 expression, and having decreased CD10 and CD20 expression levels [40]. Furthermore, CLL-1 levels do not change following therapy [40]. On the basis of these findings, CLL-1 may not be an effective indicator for ALL diagnosis, except in cases of B-ALL with DEX4 rearrangement, where CLL-1 expression can be very useful for diagnosis.

## CLL-1 in clinical setting

### CLL-1 as a prognostic/predictive marker

Acute myeloid leukemia prognosis appears to be associated with CLL-1, on the basis of the information previously mentioned. In a study published in 2017, Yan-Yu Wang et al. utilized a 42.5% cutoff expression level to divide 123 bone marrow samples from CD34 + AML patients into low and high CLL-1 expression groups [41]. These findings indicated a significant correlation between low CLL-1 expression and poor karyotype, as well as a noteworthy association with CEBPA biallelic mutation. Additionally, low CLL-1 expression was associated with a reduced likelihood of achieving complete remission after 1 cycle of induction chemotherapy, as well as decreased event-free survival and overall survival [41]. Laura Laine Herborg et al. conducted studies reviewing the prognostic impact of CLL-1, which was originally investigated by Wang et al., confirming the significance of CLL-1 as an independent prognostic parameter for overall survival (OS) in the respective selected groups. Nevertheless, these findings may not apply universally to all AML patients, emphasizing the need for further examination of LAIPs in larger cohorts at diagnosis to predict outcomes accurately [28]. In a separate investigation, Jinghua Wang et al. identified the threshold for CLL-1 expression at 59.0%, yielding an area under the curve (AUC) of 0.694 (*p* = 0.017). Afterward, the patients were divided into two high and low CLL-1 groups according to this specific point. Among the 52 samples examined, 33 were identified as high CLL-1 (> 59.0%), and 19 were classified as low CLL-1 (< 59.0%). Compared with the CLL-1-high group, the CLL-1-low group presented a significantly decreased percentage of BM blasts (*p* < 0.05) and a much lower CR rate (35.3% vs. 73.3%, *p* < 0.05). After defining CLL-1-high and CLL-1-low individuals, the significance of CLL-1 was investigated for prognosis. The group with elevated CLL-1 levels had notably greater EFS (*p* = 0.048) and OS (*p* = 0.012) than did the lower group. Moreover, the study revealed that CLL-1-high patients (9 participants) in the low-risk category had better EFS outcomes than did CLL-1-low patients (7 participants) (*p* = 0.01). Univariate analysis highlighted treatment response as the key factor influencing EFS (*p* = 0.002), whereas treatment response (*p* < 0.001) and CLL-1 expression (*p* = 0.019) were the main factors impacting OS. Multivariate analysis confirmed that treatment response and CLL-1 expression were significant independent factors for overall survival, with p values of 0.001 and 0.045, respectively. A comparison of the CLL-1high and CLL-1low groups revealed significantly lower EFS and OS in the CLL-1low group (*p* < 0.05) [29]. Although the results are noteworthy, it will be necessary to validate them in different patient populations and explore additional cutoff points for the best prognostic data. More research is needed to understand how CLL-1 expression affects AML treatment outcomes and prognosis, if at all.

### CLL-1 as a marker for monitoring AML-MRD

Owing to advancements in supportive care and the increased availability of allogenic hematopoietic cell transplantation, the outcomes for individuals with AML have gradually improved [4, 5]. Despite achieving a complete remission rate of approximately 80% and reducing the disease burden by 99.9% following initial chemotherapy induction, a proportion of residual LSCs ranging from 10^10^ to 10^12^ cells can persist [3]. Even with the implementation of consolidation therapy to eradicate persistent LSCs, over 50% of patients experience relapse or refractory disease. This is caused by the continued presence of LSCs, which cannot be identified via traditional methods (morphological examination, flow cytometry, and molecular techniques) and represent minimal residual disease (currently referred to as measurable residual disease since 2018) [3, 42]. Given the critical need for accurate and sensitive MRD detection, which remains a challenging task, significant research efforts have been directed toward devising novel approaches for precise and sensitive detection methodologies [3]. Among the rationales for the utilization of MRD detection for AML, several justifications can be delineated: (1) provide an objective way to create a deeper state of remission; (2) enhance the accuracy of predicting outcomes and guide postremission therapies; (3) serve as a predictive biomarker for enhancing the assessment of relapse risk following recovery; (4) serve as a monitoring tool for the identification of imminent relapse and facilitating prompt intervention; and (5) serve as a surrogate endpoint for expediting the evaluation and approval processes of pharmaceutical interventions [3, 11]. Regardless of hematopoietic cell transplantation, a number of studies on AML patients, both adults and pediatric patients, have demonstrated that the presence of MRD is linked to a greater risk of relapse and a shorter survival time [9, 43]. Additionally, the identification of MRD through multicolor flow cytometry immediately prior to allogeneic HCT can function as a robust and independent prognostic indicator for posttransplantation outcomes in AML patients [44, 45]. Hence, it is crucial that MRD detection techniques be reliable, sensitive, and suitable for use with all AML patients. As the most popular method of MRD detection at present, immunophenotyping is a crucial and readily available tool for AML diagnosis. In this method, ≤ 0.1% percent of CD45-expressing cells that exhibit the target immunophenotype are considered positive for MFC-MRD tests [9]. To accurately identify MRD via MFC, specialized panels targeting abnormally expressed antigens with stable expression levels during treatment but minimal presence at normal cellular levels are used [9, 11]. Notably, CLL-1 stands out as one of these antigens and is detectable on the surface of CD34 + leukemic blasts in most AML patients after initial diagnosis [33]. The consistent expression pattern of CLL-1 on leukemic blasts and LSCs at different stages—from diagnosis to treatment to relapse—underlines its reliability and robustness as a surveillance marker. In particular, the specific detection of CLL-1 expression in CD34 + CD38- AML cells after chemotherapy in AML patients who achieved complete remission highlights its prognostic value over conventional MRD detection methods [9, 33, 46]. In conclusion, the presence of CLL-1 expression on residual leukemic cells after primary therapy offers superior predictive capability for outcomes compared with MRD monitoring techniques [46]. On the basis of these studies, CLL-1 has been identified as a viable diagnostic indicator for monitoring progression and anticipating risks during the treatment of AML. Moreover, CLL-1 may be included in a single-tube approach to evaluate CD34 + CD38- leukemic stem cells, which is a procedure that the European LeukemiaNet (ELN) AML MRD 2021 consensus supports [9].

### CLL-1 as a target for the immunotherapy approaches

Despite extensive research in the area of anticancer therapy, there have been no significant breakthroughs in standard induction and consolidation treatments for AML. While approximately 10–40% of younger AML patients are resistant to induction therapy, the rate is notably greater for individuals aged 60 years and above, ranging from 40 to 60% [47]. Although most fit patients, after achieving CR, subsequently undergo hematopoietic stem cell transplantation (HSCT), 40% of these patients experience relapse following HSCT. In addition, a study by the International Center for Blood and Marrow Transplant Research (CIBMTR) revealed that only 23% of 1788 AML patients were still alive one year following posttransplantation relapse [48]. The high frequency of relapsed or refractory (R/R) AML highlights the urgent need for novel treatments to address these undesirable outcomes. Immunotherapy has gained recognition as an innovative approach for treating blood cancers and solid tumors in recent years [49]. While immunotherapy has revolutionized treatment of other cancers, it has failed to cure AML. The innate heterogeneity of AML and the challenge of identifying antigens associated with leukemic cells without causing destruction to normal hematopoiesis have collectively impeded progress in this area. Multiple studies have extensively examined many surface antigens, including CD32, CD33, CD44, CD47, CD123, and TIM-3, as potential targets for therapeutic intervention [50–55]. Nevertheless, the presence of these markers on normal HSCs is a considerable obstacle. During the past several years, immunotherapy has gained recognition as an innovative strategy for treating hematologic malignancies and solid tumors. For example, targeted therapy that employs an anti-CD33 antibody conjugated to calicheamicin (known as mylotarg) results in severe hematological toxicity [56].

To maximize therapeutic efficacy and reduce off-target toxicity, an optimal therapeutic target in AML should restrict expression within the leukemic compartment while preserving normal HSCs and progenitor cells. CLL-1 is an interesting antigen for targeted therapy since it is highly expressed on AML blasts and LSCs, which are the cells responsible for relapsing malignancy and driving disease recurrence [57]. In contrast, normal HSCs and granulocyte‒macrophage progenitors (GMPs) have restricted or absent expression of this marker. The features of CLL-1 make it an attractive candidate for immunotherapeutic interventions. Research has focused on developing medicines that specifically target CLL-1, such as monoclonal and bispecific antibodies, antibody‒drug conjugates, and chimeric antigen receptor (CAR)-modified T cells.

#### Antibody-based Therapy

**Fig. 3:**
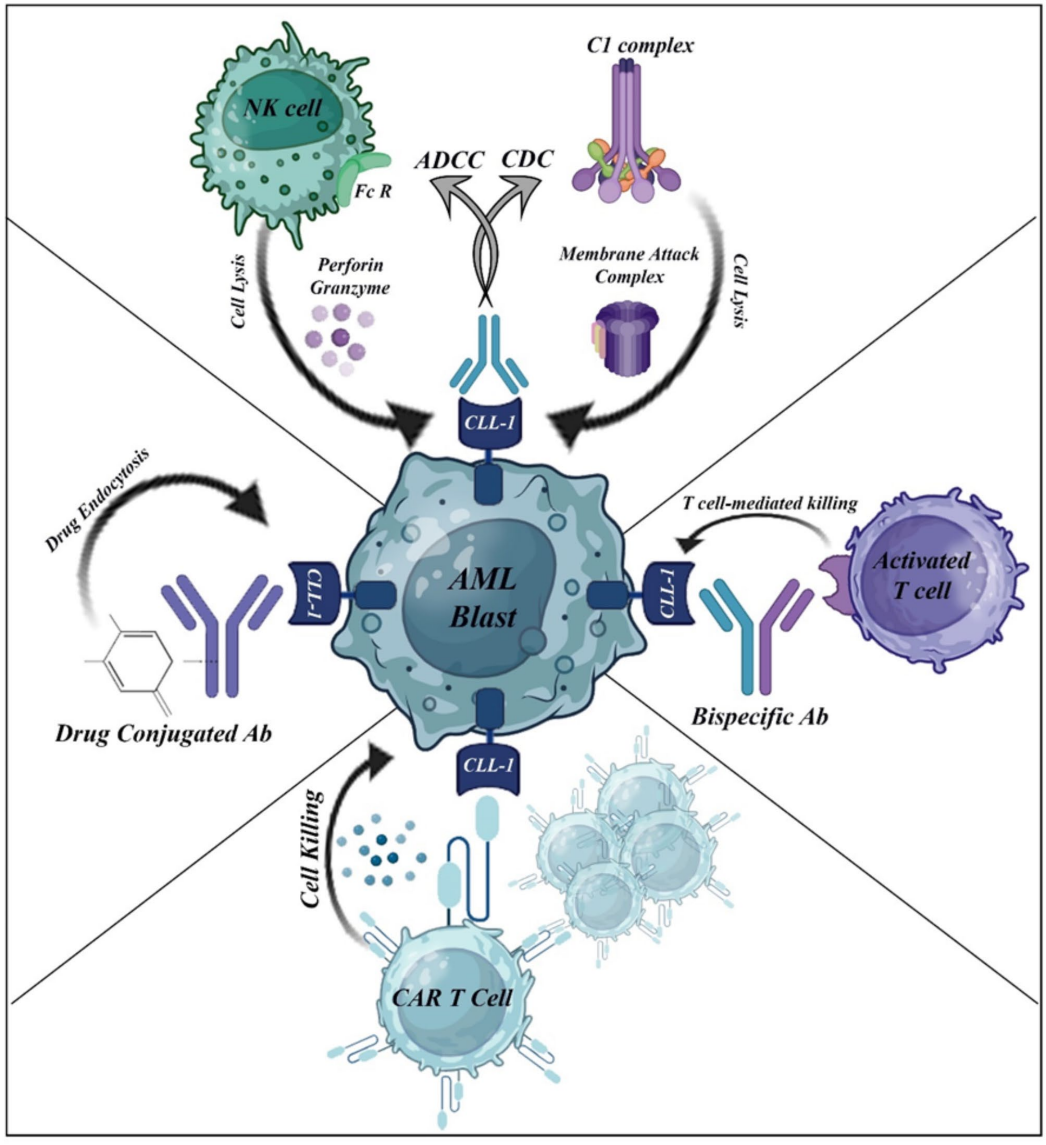
Graphical abstract the illustrating the mechanisms of immunotherapy with CLL-1 in AML blast

Intensive chemotherapy can be extremely successful for treating AML, but it also has significant adverse effects. In recent years, researchers have explored novel therapeutic options, including monoclonal antibody (mAb) therapy, which might offer a more targeted and less harmful approach to fight AML. However, the effectiveness of these therapies in eliminating AML blasts relies on their ability to stimulate certain immune responses. The main ways in which mAbs kill leukemic cells are through complement-dependent cytotoxicity (CDC) and antibody-dependent cell-mediated cytotoxicity (ADCC) (Fig. 3). Currently, the primary emphasis is on mAbs that target the CD33 and CD123 antigens in AML patients. The first phase III trial (NCT0006045) utilizing the humanized IgG1 unconjugated anti-CD33 antibody (lintuzumab) was terminated owing to its inefficacy [56]. The challenges stemmed from the low expression density in the cell membrane and the ineffective internalization of anti-CD33 antibodies. It was also shown that the unconjugated antibody (talacotuzumab) did not work to treat AML or MDS (NCT0299861). The current clinical trial (NCT04086264) is now accepting patients for a phase I/II study, including the administration of anti-CD123-DGN462, either as a single treatment or in conjunction with AZA VEN, for MRD-positive postinduction therapy patients. Recently, researchers have produced a set of mAbs that specifically target the extracellular domain of CLL-1 [58]. The mAbs exhibited potent CDC against recently obtained AML blasts, with a success rate of 94% (15 of 16 patients) in a dose-dependent manner. Researchers have shown that mAbs bind to CLL-1 can effectively internalize and shrink cancer cells in the HL-60 mice model. This implies that CLL-1 antibodies are effective in kiling leukemia cells. Nevertheless, there is a current scarcity of human studies that assess the safety and efficacy of these mAbs in patients with AML.A potential novel approach in cancer therapy is the utilization of bispecific antibodies to effectively stimulate T lymphocytes to attack target cells. Bispecific antibodies show great potential as a therapeutic approach for treating cancers that are in a dormant state, have elevated expression of drug efflux pumps, or display reduced amounts of surface antigens. The reason for this is that they function by magnifying the deadly signal produced by activated effector T cells that are specifically located in the tumor [59]. Prior studies have examined the efficacy of a bispecific antibody in treating AML by specifically targeting the CD33 antigen [60]. This bispecific antibody has modest efficacy in mice xenograft models, specifically in targeting and killing AML cell lines and primary patient samples [61]. AMG 330 was examined in a phase I trial as a bispecific T-cell engaging antibody construct targeting CD33 in patients with R/R AML [62].

Nevertheless, there has been little research conducted on bispecific antibody targets for other antigens linked with AML, including the recently discovered marker CLL-1. Targeting CLL-1 with T cells can eradicate leukemic stem cells without damaging normal HSCs because it only affects the restricted expression profile (Fig. 3). This allows for the restoration of normal hematopoiesis following treatment and possibly reduces hematological toxicity.

In this way, Lu, H. et al. described αCLL1-αCD3 BiAb, and compared its activity to that of αCD33-αCD3 BiAb [63]. Notably, αCLL1-αCD3 is 5-fold more effective than αCD33-αCD3 against all the evaluated AML cell lines, and it makes the tumor smaller in the xenograft mice model. After 10 injections, tumor cells were almost impossible to find, and the treatment had no negative effects on the mice’s health or body weight during the experiment.

MCLA-117, a CLL-1-CD3 BiFab with a native human IgG format, stimulates T-cell activation and proliferation, releases proinflammatory cytokines, and redirects T cells to lyse CLL-1-positive cells [64]. Currently, there is an ongoing clinical trial (NCT03038230) investigating the use of MCLA-117 in individuals with primary or secondary AML. The trial started in November 2019 with a total of 50 patients receiving MCLA-117, with a target dose of 0.675 to 120 mg. No dose-limiting toxicity (DLT) was identified. The recorded treatment-related adverse events (TRAEs) were nausea (10%), vomiting (12%), infusion site phlebitis (14%), chills (22%), pyrexia (32%), and cytokine release syndrome (CRS) (32%). Four of the 26 patients evaluated had a bone marrow blast decrease of more than 50%, and one of them had a morphological leukemia-free state (MLFS) [65].CD3-BiAbs that bind strongly to CD3 have significant efficacy in killing tumor cells. However, they also induce intense cytokine storms [66, 67]. To expand the therapeutic window, Leong S. et al. used a monkey cross-reactive anti-CLL-1 arm with high affinity and studied it in comprehensively with anti-CD3 arms with varying levels of affinity. They reported that the high-affinity CD3 arms were significantly more powerful in vitro—up to 100 times more effective. On the other hand, in vivo studies showed only BiAbs with low affinity for CD3/CLL1, was highly tolerated and capable of eliminating target cells. This study suggested that a properly designed CLL-1 BiAbs could be effective in treating AML [68].

To increase the effectiveness and safety of treatments for patients with AML, E Lee et al. compared two CLL-1-CD3 BiAbs in ABL602 1 + 1 and 2 + 1 forms [69]. ABL602 1 + 1 refers to a molecule with one antigen binding valency, meaning that it has an anti-CD3 Fab and an anti-CLL-1 Fab in each arm. On the other hand, ABL602 2 + 1 has an additional Fab of anti-CLL-1 attached to the N-terminal end of the anti-CD3 heavy chain linked by G4S3. Compared with ABL602 1 + 1, ABC602 2 + 1 had approximately 45-fold greater efficacy for T-cell activation, which was demonstrated by an increase in CD69+/CD8 + T cells. It effectively suppressed tumor development by stimulating the activation of T cells, even when the ratio of effector cells to target cells (E: T) was very low, at 1:50. On day 22, the groups treated with ABL602 2 + 1 presented a decrease in AML cells and increased infiltration of T cells in a dose-dependent manner. Also, ABL602 2 + 1 did not stimulate the secretion of cytokines such as IL-6 and TNF-α in peripheral blood mononuclear cells obtained from healthy donors [69].

Considering the superior safety profiles of NK cell treatments compared with those of T-cell therapies in clinical settings, a treatment based on Trispecific killer engagers (TriKEs) represents a beneficial option for bispecific T-cell engagers (BiTEs) [70]. TriKEs consist of two single-chain variable fragments (scFvs) that selectively bind to CD16a NK cell receptors and a tumor antigen. The scFv fragments are linked together with a human IL-15 moiety, creating a more sophisticated and potent therapeutic structure. CD33 TriKE is currently undergoing a phase I/II clinical trial to evaluate its effectiveness in treating patients with refractory AML and high-risk MDS (NCT03214666). Arvindam US et al. developed a CLL1-TriKE molecule that contains an anti-CD16 heavy chain that humanizes a camelid single-domain antibody (sdAb), a wild-type IL-15 moiety, and an anti-CLL1 (scFv) [71]. Treatment with the CLL-1 TriKE efficiently inhibited the NK-mediated killing of CLL-1-expressing AML cell lines, as well as primary AML blasts and LSCs, in an antigen-dependent manner. Additionally, the CLL-1 TriKE was able to decrease the tumor burden in preclinical mouse models. These findings emphasize the clinical potential of the CLL-1 TriKE for effective AML treatment. One promising approach is to combine these antibodies with chemotherapy drugs or conjugate them with toxic agents to increase their ability to fight cancer. With respect to the toxicity and off-target effects of some chemotherapy drugs, it is not possible to use these drugs as free medications in traditional chemotherapy. However, a specific benefit of using conjugated antibodies with drugs is their capacity to accurately target and eliminate cancer cells while minimizing damage to healthy cells.

#### Antibody‒drug conjugate (ADC) therapy

Antibody-drug conjugate (ADCs) has been clinically proven to improve the efficacy of induction treatment for AML by providing a very powerful cytotoxic payload that precisely targets leukemia cells while keeping systemic concentrations low. The isoquinolidinobenzodiazepine (IQB) D211 payload has cytotoxic effects on many AML cell lines. Two modified cysteine residues and a cleavable linker covalently link the D211 payload to the humanized anti-CLL-1 antibody. The selection and characterization of CLT030 (anti-CLL-1 ADC) were conducted both in vitro and in vivo, utilizing a wide range of AML cell line models and AML patient samples (Fig. 3) [72]. Additionally, CLT030 effectively suppressed tumor development in a xenograft model of AML patients. Furthermore, compared with that of CD33-ADC, the ability of normal human CD34 + cells to differentiate into diverse lineages was reduced, as revealed by both in vitro colony formation experiments and an in vivo xenotransplantation model. These findings suggest that CLL1-ADC might be a promising ADC therapy for the treatment of AML. The second ADC, DCLL9718S, is a newly developed humanized monoclonal IgG1 antibody that specifically binds to CLL-1. It is designed with two pyrrolobenzodiazepine (PBD) dimers, which function as DNA alkylating and cross-linking agents and are connected by a disulfide bond that can be cleaved. DCLL9718S is a humanized monoclonal IgG1 antibody that specifically binds to CLL-1. It is designed with two PBD dimers connected by a disulfide-based self-immolative linker, which functions as a DNA alkylating and cross-linking agent. Once DCLL9718S binds to CLL-1 on target cells, it is internalized and degraded in the lysosome. This releases the active form of the PBD dimer, which forms covalent bonds with DNA in the minor groove. This interaction causes damage to the DNA and leads to the death of the cells [73]. This study revealed that CLL-1 and CD33 have similar frequencies and trafficking properties. These findings suggest that CLL-1 could be a target for ADCs in AML. Furthermore, compared with the use of CD33, which is present on normal HSCs, the use of an anti-CLL1 ADC results in a lower level and a shorter period of bone marrow suppression. In preclinical experiments, Bing Zheng et al. demonstrated that DCLL9718S successfully eradicates tumor cells in AML xenograft models and does not exhibit target-independent toxicity at doses that deplete target myeloid cells in cynomolgus monkeys [74]. DCLL9718S entered the clinical trial process after promising results were shown in preclinical studies. Eighteen adult patients with R/R AML participated in a phase I DCLL9718S dose-escalation trial [75]. The primary objectives of the trial were to evaluate the safety and maximum tolerated dosage (MTD) of DCLL9718S. The patients were administered DCLL9718S in 21-day cycles, with a dose ranging from 10 to 160 g/kg, beginning on Day 1 of each cycle. There was no objective complete response (CR) or partial response (PR) observed in any of the eighteen patients who received DCLL9718S. The study failed to determine the MTD due to the observed hepatic injury at the highest dose and a lack of antileukemic activity. Liver damage may occur as a result of the PBD or modified PBD-like payloads, and this impact might be consistent across different classes. Owing to the insufficient tolerability and antitumor efficacy demonstrated by DCLL9718S, the program will terminate during dose escalation and not move forward in phase II trials [75].

#### CAR-modified T/NK-cell therapy

Chemotherapy and HSCT are the established therapeutic approaches for treating AML. Although there have been advancements in the treatment of AML in recent years, the 5-year survival rate is still less than 50%, mostly because of chemotherapy resistance or the toxic effects of these therapies [76]. The majority of patients ultimately die from relapse and/or progression of the disease, indicating a pressing need for new treatment approaches for these individuals. Utilization of CAR-transduced T-cell therapy is a novel and very effective technique for treating hematological malignancies. CAR-T-cell therapy synergizes the precise antigen identification ability of antibodies with the robust cytotoxic potential of T cells. Significant clinical effectiveness has been achieved in the treatment of ALL and non-Hodgkin lymphoma (NHL), which express CD19 [77, 78]. The success of CAR-T-cell therapy in treating ALL has sparked interest in its potential for the treatment of AML. However, translating this effectiveness to AML presents significant challenges. The main obstacle preventing the use of CAR-T cells in myeloid malignancies is the lack of a specialized antigen. CD33 and CD123 are currently the most common targets for CAR-T-cell therapy in AML. While AML blasts or LSCs may have higher levels of CD33 and CD123 than healthy cells do, targeting these cells still carries the risk of off-target effects, causing long-lasting or permanent myelosuppression (Table 2) [79]. However, researchers are pursuing an increasing number of antigens, including CLL-1, as targets.

**Table 2:** Comparative analysis of CLL-1 (CD371), CD33, and CD123 as therapeutic targets in AML: advantages and disadvantages

Marker	Advantage	Disadvantage
CD 33	• Expressed on the surface of most AML cells • FDA approved anti-CD33 antibody-drug conjugate (Gemtuzumab ozogamicin)	• On-target off-tumor toxicities Expressed on normal hematopoietic cells and myeloid cells • Low expression and Slow internalization Limit the activity of antibodies • High CD33-antigen load in peripheral blood • Not benefit for high-risk AML patients
CD 123	• Overexpressed in AML cells • Effectively in preclinical studies • Various CD123-targeted therapies are under investigation • Used as Marker for detection MRD	• On-target off-tumor toxicities Expression in normal tissues eosinophils, neutrophils, plasmacytoid dendritic cells • Toxicity of CD13 targeting therapies • Unsatisfactory efficacy
CD371	• Highly expressed on AML cells and leukemic stem cells (LSCs) • Various CLL-1-targeted therapies are under investigation • Low expression in another Tissue	• Expressed on normal myeloid cells, such as granulocytes and monocytes • Expression can vary among AML patients Reducing the efficacy of targeted therapies in individuals with low or absent CLL-1 expression • Limited Data on Long-Term Outcomes

Laborda E et al. created a novel CLL-1 CAR T cell that demonstrated potent and specific cytotoxicity against AML cell lines HL60, MOLM13, and MOLM14 [80]. In vivo investigations demonstrated total tumor regression, with no indications of relapse noted for up to 80 days following tumor regression and sustained presence of CAR-T cells in the xenograft HL-60 mouse model. Conversely, untreated mice succumbed to the tumor burden within three weeks [80]. Additionally, they observed no toxicity to CD34 + CD38 + cells with low levels of CLL-1 expression, suggesting that a specific threshold of CLL-1 expression is necessary for CAR-T-cell-induced lysis. The same result was observed in periclinal studies designed by Wang et al., which revealed that CLL-1 CAR-T cells efficiently destroyed U937 and HL-60 cells as well as in vivo a xenogeneic model of AML translated by U937 cells but did not have the same effect on CLL-1-negative K562 cells [80]. These CAR-T cells did not decrease the quantity of CFU-GEMM, CFU-GM, or BFU-E colonies obtained from cord blood samples [33]. These findings highlight the critical properties of this novel CAR-T-cell design for achieving durable cancer remission in a clinical setting.

Fang Liu et al. reported the initial findings of preclinical and clinical phase I trials utilizing CLL1-CD33 CAR-T cells, since CLL1 and CD33 are often employed as targets in AML treatment [81]. Preclinical investigations have provided evidence of the targeted antitumor effects of CLL-1-CD33 CAR-T cells against both cell lines and primary leukemia samples from patients with AML. Additionally, in a mouse model, CAR-T cells effectively decreased the tumor burden and prolonged survival. During the dosage escalation phase I clinical study for relapsed/refractory AML patients (NCT03795779), lymphodepletion treatment (consisting of fludarabine and cyclophosphamide) was administered to patients before CAR infusion. They administered two divided doses, with each dosage comprising 1 × 10^6^/kg CAR-T cells, on day 1 and day 2. On day 12, while the leukemia blasts still accounted for 98% of the bone marrow, there was a significant increase in the number of CAR-T cells in both the bone marrow and peripheral blood. On day 19, the patient reached MRD- status, which was fully remission, as confirmed by bone marrow aspirates showing total elimination of myeloid cells, and was permitted to proceed with allo-HSCT. One difficulty associated with CAR-T-cell therapy is the occurrence of antigen-negative relapse. For example, B-ALL patients treated with CD19 CAR-T cells relapse due to a reduction in CD19 expression on the surface of malignant cells [82]. Therefore, a strategy to address the issue of target antigen loss after CAR-T-cell therapy is to concurrently target multiple antigens. In 2023, Xie D et al. conducted preclinical research in which they developed a biscistronic CAR (123CL CAR) capable of targeting both CD123 and CLL1 to eliminate AML cells both in vitro and in vivo [83]. 123CL CAR-T cells eradicated AML cell lines and patient blasts, substantially reduced the tumor burden and improved survival rates in mouse models. To increase safety, the marker/suicide gene RQR8 was included in the CAR structure, along with the CD20-CD34 epitope. This combination allows for the detection and potential elimination of CAR-T cells that are undergoing excessive proliferation. An in vitro experiment revealed that the combination of NK cells and rituximab effectively cleared 123CL CAR-T cells through RQR8-mediated mechanisms [83]. A single-center phase I/II clinical trial is currently assessing the efficacy and safety of anti-CLL-1 CAR-T-cell therapy in four children with R/R-AML [84]. The CAR design was derived from FKBP-caspase 9, which is an apoptosis gene, to create a safer application of CAR technology, referred to as the 4SCAR. Adverse events included CRS, immune effector cell-associated neurotoxicity syndrome (ICANS), and additional adverse effects. The evaluation of treatment response was conducted via morphological and flow cytometry-based MRD assays. Notably, 3 patients achieved CR with MRD negativity. One patient had a survival duration of 5 months; however, all of these patients had mild and controllable adverse effects. These preliminary findings suggest that anti-CLL-1 CAR-T-cell therapy has the potential to be a safe and effective therapeutic option for children with R/R-AML (Tables 3 and 4) [84].

**Table 3:** CLL-1 clinical trials

Target	Phase	Study Population	Enrollment	Intervention	Status	NCT.gov identifier	Disease	
*Antibody based Therapy*								
CLL-1/CD3	Phase I	18 Years and older (Adult, Older Adult)	62 A	MCLA-117 bispecific antibody	Unknown status	NCT03038230	AML	
*CAR-modified T/NK-cell therapy*								
CLL-1	Phase I	1 Year and older (Child, Adult, Older Adult)	15 E	CD371-YSNVZ-IL18 CAR T cells	Recruiting	NCT06017258 * [86]	R/R AML- AML	
CLL-1	Early Phase 1	(Child, Adult, Older Adult)	36 E	CLL1 CAR T-cells	Recruiting	NCT05252572	AML	
CLL-1	Phase I/II	6 Years to 65 Years (Child, Adult, Older Adult)	20 E	anti-CLL1 CART	Recruiting	NCT04884984 * [87]	AML	
CLL-1	Phase I	up to 75 Years (Child, Adult, Older Adult)	18 E	CLL-1.CAR T cells	Recruiting	NCT04219163	AML	
CLL-1	Phase I	18 Years to 70 Years (Adult, Older Adult)	24 E	anti-CLL-1 CAR NK cells	Recruiting	NCT06307054	R/R AML	
CLL-1	Phase 1	2 Years to 70 Years (Child, Adult, Older Adult)	20 E	CLL1 CAR-T	Not yet recruiting	NCT05467202	AML	
CLL-1	Phase I	18 Years and older (Adult, Older Adult)	24 E	CLL1 CAR-NK cell injection	Recruiting	NCT06027853	AML	
CLL-1	Early Phase 1	2 Years to 75 Years (Child, Adult, Older Adult)	100 E	Anti-CLL1 CART cells	Recruiting	NCT04923919	AML	
CLL-1	Phase I	3 Years to 18 Years (Child, Adult)	24 E	BG1805: anti-CLL1 CAR-T cell	Not yet recruiting	NCT06347458	R/R AML	
CLL-1	Phae I/II	18 Years to 70 Years (Adult, Older Adult)	24 E	BG1805: CAR-T cell	Recruiting	NCT06118788	R/R AML	
CLL-1	Phase I	18 Years and older (Adult, Older Adult)	15 A	KITE-222, an Autologous Anti-CLL-1 CAR T-cell Therapy,	Terminated	NCT04789408	R/R AML	
C*ombination Therapy*								
*CLL-1 + CD 33*								
CLL-1/CD33	Early Phase I	(Child, Adult, Older Adult)	20 E	CLL1-CD33 CAR T cells	Unknown status	NCT03795779 * [81]	AML-MDS-MPN-CML	
CLL-1/CD33	Early Phase I	18 Years to 70 Years (Adult, Older Adult)	32 E	Drug: Fludarabine/Cytoxan Dual CD33-CLL1 CAR-T cells	Unknown status	NCT05016063	AML	
CLL-1/CD33	Early Phase 1	18 Years to 75 Years (Adult, Older Adult)	18 E	Anti-CD33/CLL1 CAR-NK Cells	Unknown status	NCT05215015	AML	
CLL-1/CD33	Phase I	1 Year to 70 Years (Child, Adult, Older Adult)	20 E	Dual CD33/CLL1 CAR T	Unknown status	NCT05248685	AML	
CLL-1/CD33	Phase I	2 Years to 70 Years (Child, Adult, Older Adult)	20 E	CLL1 + CD33 CAR-T	Not yet recruiting	NCT05467254	AML	
CLL-1/CD33	Not Applicable	1 Year to 18 Years (Child, Adult)	0	CLL1/+CD33 CAR-T	Withdrawn	NCT05943314	AML	
CLL-1/CD33	Phase I	18 Years and older (Adult, Older Adult)	24 E	iPSC -NK Cells Targeting CLL1 or CD33	Recruiting	NCT06367673	R/R AML	
CLL-1/CD33	Phase I	18 Years and older (Adult, Older Adult)	102 E	Drug: CD33/CLL1 dual CAR-NK cell Drug: Cyclophosphamide/ Fludarabine/ Cytarabine Drug: CD33 CAR-NK cell Drug: super NK cell	Not yet recruiting	NCT05987696	AML	
CLL-1/CD33	Phase I	14 Years to 60 Years (Child, Adult)	4 A	CLL1-/CD33 Targeted LCAR-AMDR Cells	Terminated	NCT05654779	AML	
*CLL-1 + CD 123*								
CLL-1/CD123	Phase II/III	up to 70 Years (Child, Adult, Older Adult)	20 E	CD123/CLL1 CAR-T Cells	Unknown status	NCT03631576	R/R AML	
*CLL-1 + CD33 + CD123*								
CLL-1/ CD33/CD123	Phase I/II	6 Months to 75 Years (Child, Adult, Older Adult)	10 E	CLL-1, CD33 and/or CD123-specific CAR gene-engineered T cells	Unknown status	NCT04010877	AML	
*Other Combination Therapy*								
CLL-1/CD38	Early phase I	18 Years to 70 Years (Adult, Older Adult)	18 E	CLL1 and CD38 dual-target CAR-T injection	Recruiting	NCT06110208	AML	
CLL-1/ CD45RA	-	18 Years and older (Adult, Older Adult)	20 E	Observational Quantification of blood cells positive for CLL1 and CD45RA surface markers by flow cytometry	Recruiting	NCT06297551 * [88]	AML	
CLL-1/ CD33/CD38/CD123	Phase I	6 Months to 75 Years (Child, Adult, Older Adult)	30 E	CLL-1, CD33, CD38 and/or CD123-specific universal CAR- T cells	Recruiting	NCT05995041	AML	
Muc1/CLL-1/ CD33/CD38/CD56/CD123	Phase I/II	2 Years to 75 Years (Child, Adult, Older Adult)	10 E	Muc1/CLL1/CD33/CD38/CD56/CD123-specific gene-engineered T cells	Unknown status	NCT03222674 * [89]	AML	

(*)The initial results of these clinical studies have been published

A: Actual Enrollment

E: Estimated Enrollment

**Table 4:** Results of clinical trials

NCT.gov identifier	Patients Enrollment	Key Findings	Date of publication	Ref
NCT03795779	a 6-year-old female patient diagnosed with a complicated karyotype AML	• Received two split doses of CLL1-CD33 CAR T cells, (1 × 10^6^/kg) • On day 19, the patient attained MRD-negative full remission, with bone marrow aspirates indicating total ablation of myeloid cells. • The patient then received nonmyeloablative HSCT • Results pertaining to other patients participating in this clinical trial, including adverse events, will be disclosed.	29, November 2018	[81]
NCT06017258	5 R/R AML adult patients	• low doses CD371-SAVVYz-IL18 CAR T cells (3 × 10^4^/kg) demonstrates significant expansion and efficacy in patients but with the risk of cytokine release syndrome (CRS) and extended cytopenia. • Prompt allo-HCT following CAR T therapy may moderate problems associated with extended cytopenia and reinforce therapeutic response.	15, November 2024	[86]
NCT04884984	2 high-risk adult AML patients, who had post-HSCT relapse and failed three lines of salvage therapy multiline salvage therapies including anti-CD38 CAR-T therapy	• Both patients achieved deep remission after CLL-1 CAR-T therapy. • Two patients receiving CLL-1 CAR-T therapy only suffered a reversible grade 1–2 CRS, without ICANS and off-target effects. • They experienced prolonged myelosuppression and showed persistent thrombocytopenia.	15, February 2022	[87]
NCT06297551 Prospective study	7 patients with newly diagnosed or relapsed AML	• A Signiant decrease in CLL1 + or CD45RA + LSC/HSC subsets in PB was observed as early as Days 5 to 7 of induction therapy • This decrease in LSC/HSC subset biomarkers appears to be associated with complete remission by the end of the treatment cycle.	5 November 2024	[88]
NCT03222674	7 patients • 4 patients received CD28/CD27-modified anti-CLL1 CAR T-cells • 3 patients were administered 4-1-BB-modified anti-CLL1 CAR T-cells.	• Five patients attained CR, with three exhibiting negative and two exhibiting positive MRD. • The total response rate was 75% (3/4) in the CD28/CD27 group and 67% (2/3) in the 4-1-BB group, respectively.	9 April 2023	[89]

Recently, a phase I clinical trial of CLL-1 CAR-T cells was conducted in a cohort of 10 adult patients diagnosed with R/R AML [85]. Among all patients, the average expression rate of CLL-1 in the membrane of tumor cells was 85.2%, with a range of 50.2–97.6%. The administered dosage of CAR-T cells ranged from 1 to 2 × 10^6^/kg, with a median of 1.5 × 10^6^/kg. During infusion, the majority of patients experienced fever, which is presumed to be an infusion-related response and is not associated with CRS. However, fever typically manifests within the 4–14-day timeframe after CAR injection, which is directly associated with the onset of neutropenia. While none of the 10 patients developed CRES, corticosteroids and tocilizumab were used to manage the development of CRS in all patients. Nevertheless, all patients exhibited severe pancytopenia; 9 patients experienced grade 3–4 agranulocytosis, 7 patients experienced grade 3–4 anemia, and 7 patients experienced grade 3–4 thrombocytopenia. Among the 10 patients, 7 achieved CR/CRi, and 6 patients were alive at the end of the entire 173-day follow-up period, ranging from 15 to 488 days (Fig. 3) [85].

## Limitations & challenges in CLL-1 research and clinical applications

Although CLL-1 has emerged as a promising target in AML therapy, significant gaps remain in our understanding of its clinical utility. Variability in CLL-1 expression across different AML subtypes and patients complicates its universal application, highlighting the need for further research to determine which patient populations would benefit most from CLL-1-targeted therapies. The inconsistency of CLL-1 expression between different leukemic blasts and in the bone marrow microenvironment raises concerns about the reliability and predictability of therapeutic outcomes.

Furthermore, long-term clinical data is lacking to evaluate the efficacy and safety of CLL-1-targeted therapies, such as CAR-T cells and antibody-drug conjugates. These therapies hold great promise but require robust, multicenter clinical trials to assess their true potential in diverse patient cohorts and across different stages of AML. Another critical issue is the potential for antigen escape mechanisms, such as loss of CLL-1 expression or downregulation, which could undermine the durability of these therapies and contribute to relapse, a major obstacle in AML treatment.

In addition, while CLL-1 is a promising marker for MRD monitoring, the absence of standardized protocols and validation across diverse patient cohorts limits its widespread adoption in clinical practice. The lack of a uniform definition of what constitutes positive or negative MRD status for CLL-1 also hinders its use as a reliable biomarker for monitoring therapeutic efficacy and disease progression.

Future studies should concentrate on creating standardized diagnostic tests for CLL-1 expression, refining treatment approaches to reduce antigen escape, and conducting extensive multicenter trials to assess the long-term safety and effectiveness of CLL-1-based treatments to fill these gaps.

## Conclusion

The emergence of CLL-1 as a promising therapeutic target in AML signifies a critical advancement in the quest for more effective and precise treatments. Its unique expression profile on AML blasts, coupled with minimal presence on normal hematopoietic cells, establishes it as an attractive candidate for targeted therapies. Preclinical investigations have demonstrated the potential of CLL-1-directed immunotherapeutic strategies, such as CAR-T-cell therapy, antibody-drug conjugates (ADCs), and bispecific antibodies. Despite these advances, several challenges persist, necessitating further exploration and refinement to optimize the clinical utility of CLL-1.

To fully realize the therapeutic promise of CLL-1, a multifaceted research approach is imperative. First, combination strategies should be explored to assess the synergistic effects of CLL-1-targeted therapies with existing AML treatments, including chemotherapy and other immunotherapies, to enhance efficacy and mitigate resistance. Second, leveraging the unique expression profile of CLL-1 for improved biomarker development can refine its role in AML prognosis and MRD monitoring. Third, well-designed clinical trials are crucial to evaluate the long-term safety, efficacy, and patient outcomes of CLL-1-directed therapies across diverse AML subtypes, thereby providing robust clinical evidence. Addressing patient-specific variability in CLL-1 expression represents another critical area, as it could help develop personalized therapeutic approaches and reduce treatment failures in cases of low or absent CLL-1 expression. Lastly, exploring advanced therapeutic modalities, such as trispecific antibodies and CAR-NK cell therapies, holds promise for maximizing therapeutic efficacy while minimizing off-target effects.

## Data Availability

No datasets were generated or analysed during the current study.
